# Synthesis, Bioevaluation and Structural Study of Substituted Phthalazin-1(2*H*)-ones Acting as Antifungal Agents

**DOI:** 10.3390/molecules18033479

**Published:** 2013-03-18

**Authors:** Marcos Derita, Esther del Olmo, Bianca Barboza, Ana Esther García-Cadenas, José Luis López-Pérez, Sebastián Andújar, Daniel Enriz, Susana Zacchino, Arturo San Feliciano

**Affiliations:** 1 Pharmacognosy Department, Faculty of Biochemical and Pharmaceutical Sciences, National University of Rosario, Suipacha 531, 2000 Rosario, Argentine; E-Mail: mgderita@hotmail.com (M.D.); 2 Department of Pharmaceutical Chemistry, Faculty of Pharmacy, CIETUS-IBSAL, University of Salamanca, Campus Miguel de Unamuno, 37007 Salamanca, Spain; E-Mails: bianca.barboza@gmail.com (B.B.); anaester@usal.es (A.E.G.-C.); lopez@usal.es (J.L.L.-P.); asf@usal.es (A.S.F.); 3 Faculty of Chemistry, Biochemistry and Pharmacy, National University of San Luis (UNSL), Chacabuco 917, 5700 San Luis, Argentine; E-Mails: saanduja@unsl.edu.ar (S.A.); denriz@unsl.edu.ar (D.E.); 4 IMIBIO-CONICET, Universidad Nacional de San Luis, Chacabuco 915, 5700 San Luis, Argentina

**Keywords:** benzylphthalazinones, antifungal, *Cryptococcus neoformans*, dermatophytes structure–activity relationships, conformational study

## Abstract

Twenty-five polysubstituted phthalazinone derivatives were synthesized and tested for their antifungal activity against a panel of pathogenic and clinically important yeasts and filamentous fungi. Among them, the compound 4-(4-chlorobenzyl)-2-methylphthalazin-1(2*H*)-one (**5**) exhibited a remarkable antifungal activity against standardised strains of dermatophytes and *Cryptococcus neoformans*, as well as against some clinical isolates. A physicochemical study performed on compound **5** revealed its conformational and electronic characteristics, providing us with useful data for the future design of novel related antifungal analogues.

## 1. Introduction

Fungal infections have emerged as a major cause of morbidity and often of mortality in immunocompromised and debilitated patients over the past decades. A matter of concern in the treatment of fungal infections is the limited number of efficacious antifungal drugs available [1,2]. Many of the currently available drugs are toxic, produce recurrence or lead to the development of resistance, due in part to the prolonged periods of drug administration needed [3]. Although a new generation of triazoles, polyenes in lipidic formulations and echinocandins have been introduced, and several combination therapies have been configured as therapeutic alternatives during the last decade, fungal infections remain difficult to eradicate [3]. There is, therefore, a clear need of discovering new structures with antifungal properties, that could lead to the development of new useful agents for the management of fungal infections.

In the course of our on-going screening program for new and selective antifungal compounds, we have previously reported several series of antifungal compounds obtained from natural and synthetic sources [4,5,6,7,8,9]. Considering that some phthalazine derivatives, including some polybrominated compounds [10], 4-benzyl substituted ones [11] and others [12,13,14,15] have been evaluated for their antimicrobial and particularly antifungal activities against yeasts (*Candida* and *Cryptococcus* strains) and *Aspergillus* spp., we have prepared a series of twenty five differently substituted phthalazin-1-ones to evaluate their antifungal activities against a panel of representative clinically important fungal species. Then, taking into account the antifungal results, conformational and electronic studies on the most interesting compound of the series were carried out.

## 2. Results and Discussion

### 2.1. Chemistry

**Scheme 1:**
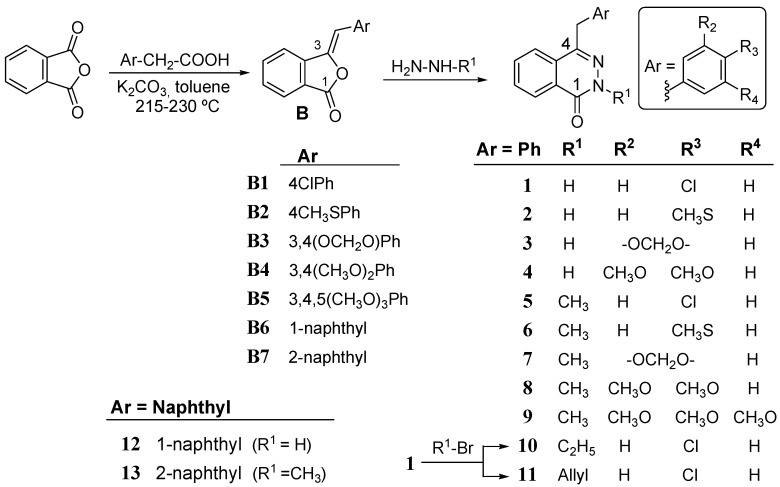
Synthesis and structures of benzalphthalides **B1**–**B7** and phthalazinones **1**–**13**.

A first group of phthalazinones **1**–**13** (Scheme 1) was synthesized from the intermediate 4-benzalphthalides **B1**–**B7** by treatment with either hydrazine or methyl hydrazine. Previously, the benzalphthalides were prepared in usually good though variable yields (90–45%) by high temperature condensation of phthalic anhydride with mono-, di- or tri-substituted phenylacetic acids, in the presence of toluene and potassium carbonate following a reported procedure [16], with a slight variation. The condensation of benzalphthalides **B1** to **B4** and **B6** with hydrazine hydrate at 80 °C during 6–8 h yielded the phthalazinones **1**–**4** and **12** respectively, while the reaction of benzalphthalides **B1** to **B5** and **B7** with methylhydrazine under the same conditions gave phthalazinones **5**–**9** and **13**, respectively.

According to our preliminary evaluation results of this first group of phthalazinones, which will be described below, the presence of the 4-chlorobenzyl substituent at position C-4 was considered as the most relevant feature for the antifungal activity. Consequently, such a moiety was maintained in the compounds synthesized later. Similarly, phthalazinones without a methyl group at the N-2 position failed to show any noticeable antifungal activity (MIC values > 250 μg/mL), whereas the *N^2^*-methylated analogues displayed from fair to good inhibition results. In the continuation of the research, the change of the methyl group at position N-2 of the phthalazinone for ethyl or allyl groups led to compounds **10** and **11**, respectively. These compounds were synthesized through direct alkylation of the phthalazinone **1** with the corresponding alkyl or alkenyl bromide. Once evaluated, the N^2^-ethyl derivative was less active and less potent than the *N*-methyl analogue, and the *N*-allyl derivative resulted practically inactive. These observations influenced the criteria applied further in this research.

**Scheme 2:**
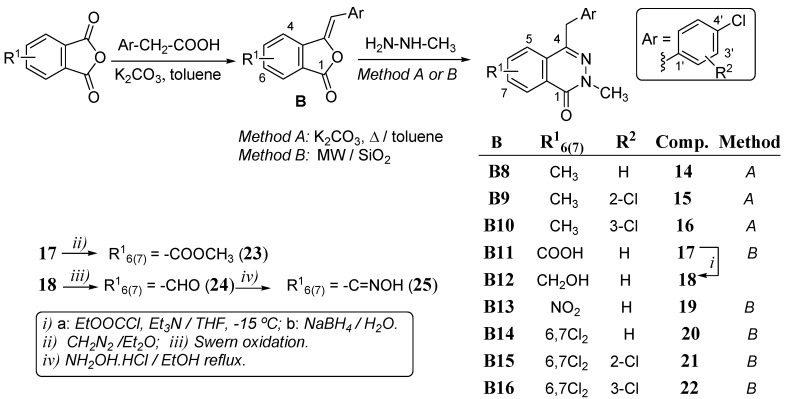
Synthesis and structures of benzalphthalides **B8**–**B16** and phthalazinones **14**–**25**.

Thus, the next step was focused to the introduction of structural modifications on the aromatic ring of the starting phthalic anhydride, while retaining the 4-chlorobenzyl fragment at C-4 and the methyl group at N-2. The modifications of the phthalazine system included the introduction of substituents with electron donating (Me), withdrawing (Cl) and with extended resonance (NO_2_) properties. The preparation of phthalazinones **14**–**25** was carried out by the procedures represented in Scheme 2. In several cases, a microwave (MW)-based procedure (method B) applied to improve reaction times and yields, also led to cleaner reaction products. The intermediate benzalphthalides **B8**–**B16** were previously prepared by the procedure mentioned above. The benzalphthalides monosubstituted on the phthalazine system **B8** to **B11** and **B13** were obtained as 1:1 mixtures of regioisomers with the substituent indistinctly attached at positions C-5 or C-6 of the benzalphthalide. The benzalphthalide **B12** was obtained by sodium borohydride reduction of the mixed anhydride intermediate obtained by treatment of **B11** with ethyl chloroformate in THF at low temperature (−15 °C), in the presence of triethylamine (TEA).

Phthalazinones **14**–**17** and **19**–**22** were obtained in good yields by treatment of the corresponding benzalphthalides with methyl hydrazine at 80 °C, during 6–8 h. The phthalazinone **23** was obtained from phthalazinone **17** after treatment with diazomethane. The phthalazinone-aldehyde **24** was obtained from the 6(7)-hydroxymethylphthalazinone **18** under Swern oxidation conditions. Finally, the treatment of aldehyde **24** with hydroxylamine under reflux in ethanol yielded the phthalazinone **25** in good yield. Phthalazinones **20**–**22** were obtained by irradiation in a domestic multimode microwave (MW) apparatus. Equimolar amounts of benzalphthalides **B14**–**B16** and methylhydrazine were mixed with SiO_2_ (10 mol) and irradiated at 350 W during 1–6 min, the mixture was percolated with ethyl acetate and the crude purified by column chromatography to provide the desired phthalazinones **20**–**22** in 60–70% yield. It is interesting to note the advantages of the MW-based procedure that led to cleaner reactions products in these cases and have previously served to prepare different phthalazine derivatives [17]. Indeed, when method A was applied to the dichlorinated benzalphthalides **B14**–**B16** more complex reaction mixtures were obtained, in which, apart from the expected phthalazinones **20**–**22**, in lowered yields, several compounds (not reported here) derived from chlorine substitution by methylhydrazinyl groups were also found.

### 2.2. Antifungal Activity

The phthalazinone derivatives included in this research were tested in the range from 250 to 0.98 μg/mL against a panel of clinically important fungi including yeasts, hyalohyphomycetes and dermatophytes with the microbroth dilution method according to the CLSI guidelines [18,19]. Results against yeasts showed that none of the compounds inhibited the yeasts *Candida* *albicans*, *Saccharomyces cerevisiae* or the *Aspergillus* species filamentous fungi *A. niger*, *A. fumigatus* or *A. flavus*, with the exception of compound **5** that inhibited the standardized strain *Cryptococcus neoformans* ATCC 32264. In contrast, ten out of the twenty five phthalazinones tested (compounds **5**–**10**, **14**–**16**, **21**) showed good to moderate activities against the dermatophytes *Microsporum canis* *(M.c.)*, *Microsporum gypseum* *(M.g.)*, *Trichophyton mentagrophytes* *(T.m.).*, *Trichophyton rubrum* *(T.r.)* and *Epidermophyton floccosum* *(E.f.)*, being also compound **5** the most active substance (Table 1 and Table 2). 

**Table 1:** Antifungal activity (MIC values, μg/mL) of phthalazinones **1**–**13** against dermatophytes.

Comp.	*E.f.*	*M.c.*	*M.g.*	*T.r.*	*T.m.*
1	*i*	*i*	*i*	*i*	*i*
2	*i*	*i*	*i*	*i*	*i*
3	*i*	*i*	*i*	*i*	*i*
4	*i*	*i*	*i*	*i*	*i*
5	6.25	6.25	25	12.5	25
6	250	100	100	125	50
7	100	125	*i*	100	*i*
8	125	125	*i*	*i*	62.5
9	*i*	*i*	*i*	100	125
10	50	62.5	50	50	50
11	*i*	*i*	*i*	*i*	*i*
12	*i*	*i*	*i*	*i*	*i*
13	*i*	*i*	*i*	*i*	*i*
AmB	0.075	0.50	0.125	0.075	0.075
Terb	0.04	0.04	0.04	0.01	0.025

*i*: Compound considered inactive (MIC > 250 μg/mL); AmB: Amphotericin B; Terb: Terbinafine; *E f.* = *Epidermophyton floccosum*; *M.c. = Microsporum canis*; M.g. = *M. gypseum*; *T.r.* = *Trichophyton rubrum*; *T.m.= T. mentagrophytes.*

**Table 2:** Antifungal activity (MIC values, μg/mL) of phthalazinones **14**–**25** against dermatophytes.

Comp.	*M.c.*	*M.g.*	*T.r.*	*T.m.*
5	6.25	25	12.5	25
14	*i*	125	100	50
15	*i*	250	50	100
16	*i*	*i*	100	100
17	*i*	*i*	*i*	*i*
18	*i*	*i*	*i*	*i*
19	*i*	*i*	*i*	*i*
20	*i*	*i*	*i*	*i*
21	*i*	*i*	*i*	*i*
22	*i*	*i*	*i*	*i*
23	*i*	*i*	*i*	*i*
24	*i*	*i*	*i*	*i*
25	*i*	125	100	100
AmB	0.50	0.125	0.075	0.075
Terb	0.04	0.04	0.01	0.025

*i*: Compounds considered inactive (MIC > 250 μg/mL); AmB: Amphotericin B; Terb: Terbinafine; *E.f.* = *Epidermophyton floccosum*; *M.c. = Microsporum canis*; M.g. = *M. gypseum*; *T.r.* = *Trichophyton rubrum*; *T.m.= T. mentagrophytes.* **^§^** Compounds with only one substituent at position 6 (7), actually contain 1:1 mixtures of both regioisomers.

#### 2.2.1. Analysis of the Activity against Dermatophytes

Table 1 summarizes the results of the antifungal activity found for phthalazinones **1**–**13**, all of which possess no substituent at the fused benzene ring of the phthalazine system. As it can be seen, the phthalazinone derivatives **1**–**4** and **12**, without substitution at N-2, were inactive (MIC values > 250 μg/mL). The comparison between those 2-methyl compounds **1**, **5**, **10** and **11**, easily led us to define the Me group as the best substituent at N-2, within those compounds tested. However, it is noteworthy that the N-Me substitution is not by itself sufficient for phthalazinones to display antifungal activity, since a change of the substituent at C-4 (benzyl to 2-naphthylmethyl), led to compound **13** which is devoid of antifungal activity. At this respect, another fact that can be observed when comparing the results related to the absence or presence of a 4-chlorobenzyl substituent at C-4, that seems to be determinant for the activity and is present in the two most potent compounds of this group, **5** and **10**. Accordingly, the concurrence of both substituents, Me on N-2, and Cl at the *p*-position of benzyl group, would be the structural features that combine for the antifungal properties of compound **5**.

Other substituents (MeS-, -OCH_2_O-, MeO-) on the benzyl side chain along with N-Me, provide the antifungal phthalazinones **6**–**9**, which showed just moderate activity. In addition, the comparison of antifungal potencies of compounds **1** *vs.* **5**, **2** *vs.* **6**, **3** *vs.* **7** and **4** *vs.* **8** showed that the different substituents at the *p*-position of the benzyl moiety need to be accompanied by an N-Me group to show antifungal activity. 

The interesting antifungal activities of compound **5** led us to prepare the analogues **14**–**25**, all of them containing both a Me substituent at N-2 and the 4-chlorobenzyl fragment at the C-4 position. These compounds were evaluated against the complete panel of fungi, though only positive results are included in Table 2.

Activity results in Table 2 show that the introduction of a methyl substituent at C-6(7) (compounds **14**–**16**) rendered compounds with 2–10 times lower antifungal activity than compound **5** and a narrower spectrum of action. Interestingly enough, the change of the Me on C-6(7) to a variety of electron-withdrawing groups as COOH, COOMe, CH_2_OH, NO_2_ or CHO (compounds **17**–**19** and **23**–**24**), or even the introduction of two chlorine substituents on C-6 and C-7 (compounds **20**–**22**) led to inactive compounds. However, compound **25** with a hydroxylimino function at positions C-6(7), and the 4-chlorobenzyl group at C-4, showed moderate activity against three dermatophyte strains.

We note also that the addition of an extra chlorine substituent at either position 2' or 3' of the benzyl fragment attached to C-4 of the phthalazinone system in compounds **15**, **16**, **21** and **22** did not produce significant changes in the antifungal activity in comparison with their respective monosubstituted 4-ClBn analogues **14** and **20**. 

#### 2.2.2. Analysis of the Activity against Yeasts

Results against yeasts showed that compound **5** was the only one that showed antifungal activity in at least one yeast (*C. neoformans*) of the panel with a value of MIC = 12.5 µg/mL. *C. neoformans* remains as an important life-threatening complication for immunocompromised hosts, particularly for patients who have undergone transplantation of solid organs. The seriousness of this pathogenic yeast has increased in the last decade, because of the appearance of fluconazole-resistant *Cryptococcus* strains. Consequently, new compounds acting against this fungus are highly desirable [20,21]. Therefore, we decided to test compound **5** against an extended panel of *C. neoformans* clinical isolates provided by the Malbrán Institute (MI, Buenos Aires, Argentina). The results are shown in Table 3. For the sake of comparison the MIC and Minimum Fungicidal Concentration (MFC), values found against an ATCC standardized strain of *C. neoformans* are included. MIC values were determined against this new panel by using three endpoints: MIC_100_, MIC_80_ and MIC_50_ (the minimum concentration of compounds that inhibit 100, 80 and 50% of fungal growth, respectively). The application of less stringent endpoints such as MIC_80_ and MIC_50_ has been shown to represent the *in vitro* activity of compounds more consistently [22] and many times provides a better correlation with other measurements of antifungal activity [23]. The evaluation of the MFC for compound **5** was accomplished by sub-culturing a sample of culture medium from MIC tubes showing no growth, onto drug-free agar plates.

**Table 3:** Minimum Inhibitory Concentration (MIC) and Minimum Fungicidal Concentration (MFC) values of phthalazinone **5** against clinical isolates of *Cryptococcus* *neoformans*.

Strain	Voucher specimen	Phthalazinone 5				AmB	Itz	Vcz
		MIC_100_	MIC_80_	MIC_50_	MFC	MIC_100_	MIC_100_	MIC_100_
*C. neoformans*	ATCC 32264	7.8	7.8	3.9	15.6	0.25	0.15	<0.015
*C. neoformans*	IM 983040	3.9	3.9	3.9	7.8	0.13	<0.015	<0.015
*C. neoformans*	IM 972724	3.9	3.9	3.9	15.6	0.06	0.25	<0.015
*C. neoformans*	IM 042074	15.6	7.8	3.9	31.3	0.25	<0.015	<0.015
*C. neoformans*	IM 983036	*i*	*i*	*i*	*i*	0.25	<0.015	<0.015
*C. neoformans*	IM 000319	125	62.5	31.3	250	0.13	<0.015	<0.015
*C. neoformans*	IM 972751	62.5	62.5	31.3	250	0.25	<0.015	<0.015
*C. neoformans*	IM 031631	62.5	31.3	15.6	125	0.25	<0.015	0.03
*C. neoformans*	IM 031633	15.6	7.8	7.8	31.3	0.13	0.25	0.25

MIC_100_, MIC_80_ and MIC_50_: concentration of compound **5** (μg/mL) that inhibits 100, 80 or 50% the control growth respectively. ATCC: Voucher specimen from American Type Culture Collection (Manassas, Virginia, USA); IM: specimens from the Malbrán Institute (Buenos Aires, Argentina). AmB = Amphotericin B; Itz = Itraconazole; Vcz = Voriconazole; *i*: MIC ≥ 250 µg/mL.

Results in Table 3 showed that **5** was fungicidal rather than fungistatic against seven out of the eight clinical isolates. It displayed strong antifungal activity (MIC50 and MIC80 between 3.9 and 15.6 µg/mL) against five out of the eight clinical isolates tested, and showed lower but still significant activity against the rest of the isolates. Although MIC values of the reference drugs amphotericin B, itraconazole and voriconazole against *Cryptococcus neoformans* are considerably lower than those displayed by compound **5**, it is worth to take into account that five or six MIC100, MIC80 or MIC50 values found for this compound against the nine fungal strains tested (Table 3), were lower than 20 µg/mL, which is indicative of a high antifungal potency.

### 2.3. Conformational and Electronic Study of Compound **5**

**Figure 1:**
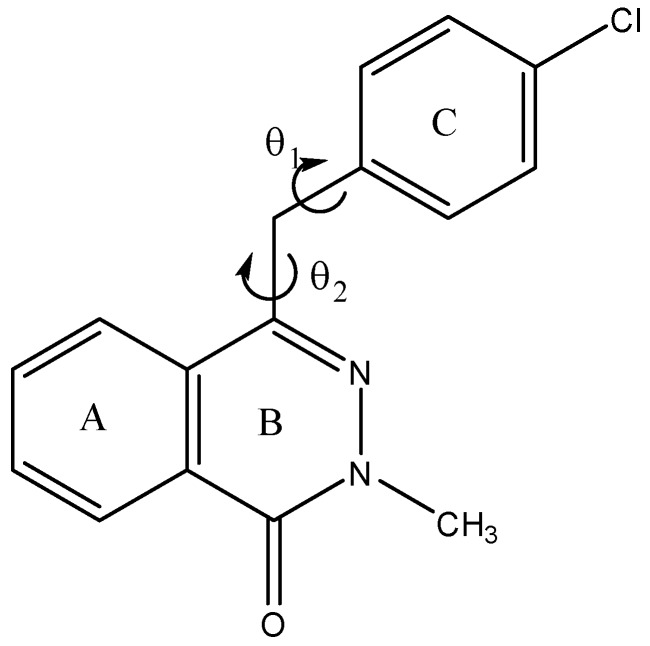
Phthalazinone 5 with definition of rings and main torsional angles.

With the purpose of obtaining a better structural information, and aiming to facilitate future design of better drugs in this field, we conducted a computer-assisted conformational and electronic study on compound **5** focused on its spatial orientations and electronic distribution. Compound **5** looks like a simple conformational problem with mainly two torsional angles (θ_1_ and θ_2_, Figure 1). For the sake of clarity, we have given the names A, B and C to the three rings of the whole molecule.

**Figure 2:**
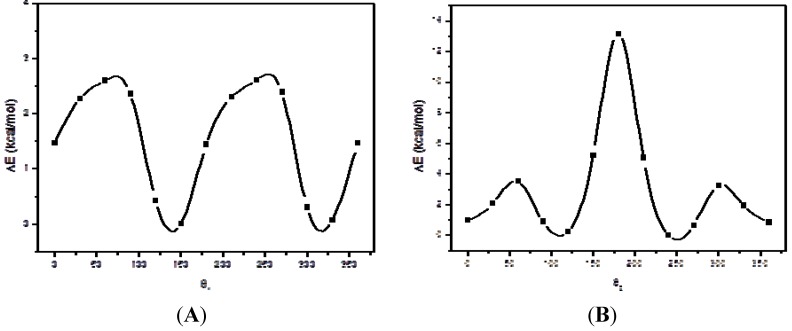
Potential Energy Curves (PECs) obtained for torsional the angles θ_1_ and θ_2_ φ1 of compound **5**. The curves were calculated at B3LYP/6-31G(d,p) level of theory.

In a preliminary and exploratory step, the conformational study of this molecule was carried out from a double scan of θ1 *vs.* θ_2_ using semiempirical PM6 calculations. To obtain such a surface we rotated the torsional angle θ_1_ *vs.* θ_2_ each 20°. PM6 calculations predict that the conformationally allowed space for compound **5** is somewhat restricted. In this surface, we observed four conformational allowed zones; however, we noted that this compound possesses at least four equivalent conformers. The surface also suggests that the planar conformations possessing θ2 ≈ 180° display very high energies. Although the semi-empirical calculations can define broad conformational features, one should employ a more accurate method, such as DFT calculations to ensure that the molecular flexibility and relative stability of the conformers are correct. Thus, we performed B3LYP/6-31G(d,p) optimizations in order to confirm the preliminary results obtained from PM6 calculations. DFT optimizations confirm the semiempirical calculations giving four energetically equivalent conformations for this molecule. These preferred form displayed half-extended conformations. The conformational analysis of compound **5** requires, at this point, the evaluation of the flexibility, *i.e.*, the energy determination of the transitional barrier between the predicted conformers. This is of crucial importance because, if the barriers are low, during a molecular recognition, this compound could be converted, with a low energy cost, to the preferred form. Energy profiles of compound **5** obtained from B3LYP/6-31G(d,p) calculations are given in Figure 2(A and B), which show the influence of ring orientations on the potential energy of the rotamers. To understand the significance of the rotation barrier, it is important to look not just to the magnitude of the energy barriers, but also to the complete behaviour energy *vs* rotation angle. Figure 2(A) shows that B3LYP/6-31G(d,p) calculations predict two conformations for θ1, those with θ1 near to 130° and 330°. We obtained barriers of about 2.5 Kcal mol^−1^ for the conformational interconversion at DFT level, indicating a significant molecular flexibility for this rotation.

In turn, Figure 2B shows the rotational behaviour obtained for the torsional angle θ2. In this case, conformations near to 0.0°, 120.0° and 240° are the preferred forms, whereas the planar form possessing θ2 near to 180° is a markedly disfavoured conformation due to the steric hindrance. For this torsion, the barrier for the interconversions is somewhat higher (3.8 Kcal/mol) than that obtained for θ1. From these results, we can conclude that the molecular flexibility of this compound is significant but moderate.

**Figure 3:**
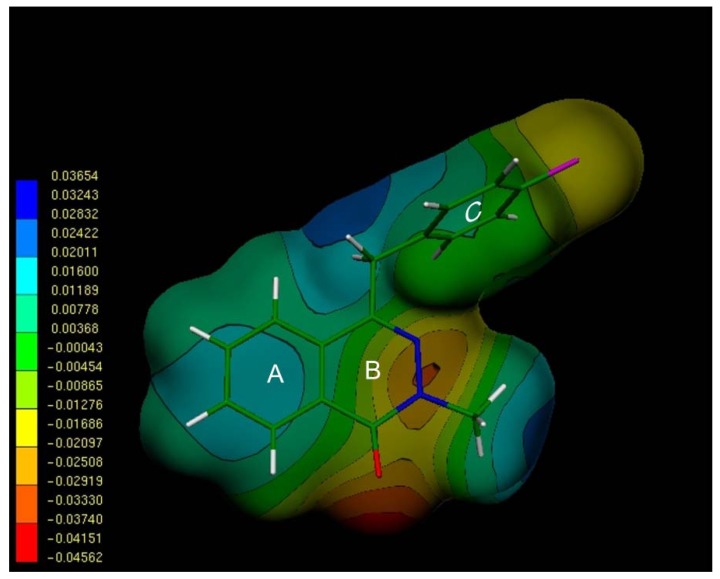
Electrostatic potential-encoded electron density surface obtained for compound **5**.

Once obtained the energetically preferred form of compound **5**, then we performed an electronic analysis using molecular electrostatic potentials (MEPs). Figure 3 shows the MEPs obtained for the preferred conformation of compound **5**. The MEP map of this molecule exhibited three clear minima, one deep red zone located in the proximity of the carbonyl group (V_(r)_ of about −0.045 el/au^3^), a second minimum in the vicinity of the N atom (orange zone, V_(r)_ of about −0.025 el/au^3^). Near to the ring C we observed a relatively extended hydrophobic zone (yellow and green area with V_(r)_ ranging from −0.02 to 0.008 el/au^3^). This third minimum correspond to ring C and from the V_(r)_ values obtained for this zone it is evident that the presence of a chlorine substituent at *p*-position of ring C polarizes this ring. We consider that, despite its symmetrical nature, this aromatic ring could make a specific contribution to the binding *via* its particular aromatic ring orientation. Thus, considering our experimental results, it appears that the presence of a chlorine substituent at the *p*-position at ring C could be important for attaining such an interaction. In this sense, the stereoelectronic changes induced by the presence of an additional chlorine atom, the common feature of many synthetic antifungal drugs, at the *ortho* or *meta* positions, could be the reason of the decreased activity found for the dichlorobenzyl derivatives **15**, **16**, **21** and **22** in comparison with **5**.

Predictions of ADME, absorption and distribution parameters and the calculated physicochemical properties (log S = −4.4, clog P = 3.7 ) for compound **5** and its analogues, are within the typical ranges desired for a drug, as well as the fulfillment of Lipinski's rule permit us to consider this substance as a good lead compound for antifungal activity.

The surface was generated with GAUSSIAN 03 using a B3LYP/6-311++G(d,p) single point calculation. The colouring represents electrostatic potential with red indicating the strongest attraction to a positive point charge and blue indicating the strongest repulsion. The electrostatic potential is the energy of interaction of the positive point charge with the nuclei and electrons of a molecule. It provides a representative measure of overall molecular charge distribution. The colour-coding is shown on the left.

## 3. Experimental

### 3.1. Chemistry

Melting points (mp) were determined in a Büchi apparatus in open capillaries and were uncorrected. All commercial chemicals were used as purchased and solvents purified by the standard procedures prior to use [24]. Thin-layer chromatography was performed on Merck 60 silica gel GF-254 precoated plates and the identification was done with UV light and colorization with 10% phosphomolybdic acid or ninhydrin spray followed by heating. Flash column chromatography was performed on Merck 60 silica gel (0.063–0.2 mesh). Infrared spectra were recorded using neat samples, without solvent or KBr, on a FT-IR spectrometer Nicolet Impact 410 model. NMR spectra were recorded on Bruker AC 200 (200 MHz) and Bruker DRX 400 (400 MHz) instruments. Chemical shifts (δ) are expressed in parts per million (ppm) relative to the residual solvent peak: CDCl3 7.26 ppm/77.0 ppm and coupling constants (*J*) are reported in Hertz (Hz). High-resolution mass spectra (HRMS) were recorded on a QSTAR XL mass spectrometer, by electron spray ionisation (ESI-MS) technique (5 kV).

#### 3.1.1. General Procedure for the Synthesis of Benzalphthalides **B1**–**B16**

Phthalic anhydride (2.2 mmol), the corresponding phenylacetic (naphthylacetic) acid (2.7 mmol), sodium acetate (0.26 mmol) and toluene (5 mL) were placed in a round-bottom flask to which a Dean-Stark separator was adapted. The mixtures were maintained at 210–245 °C under nitrogen and with magnetic stirring for 9–33 h. After cooling, the reaction mixtures were dissolved with ethyl acetate and washed with aqueous Na_2_CO_3_ (sat.), brine and water, dried over Na_2_SO_4_ and concentrated under reduced pressure to give the crude reaction products. Solid products were purified by crystallization and oily products chromatographed over silica gel; yields ranged from 40–95%. All the benzalphthalides were obtained as the *Z* isomer, and the configuration was confirmed through NOE-difference and/or 2D-ROESY experiments.

*(Z)-3-(4-Chlorobenzylidene)isobenzofuran-1-one* (**B1**). Yield 75%. Yellow crystals; mp 172–174 °C; IR (KBr), ν_max_: 2919, 1796, 1656, 1450, 1366, 1270, 1078, 969, 850, 825, 758, 606 cm^−1^. ^1^H-NMR δ: 6.30 (s, 1H, H-8), 7.30 (d, *J* = 8.8 Hz, 2H, H-3'+ H-5'), 7.53 (d, *J* = 7.8 Hz, 1H, H-4), 7.54 (m,1H, H-6), 7.68 (m, 1H, H-5), 7.70 (d, *J* = 8.8 Hz, 2H, H-2'+ H-6'), 7.87 (d, J = 7.8 Hz, 1H, H-7) ppm. ^13^C-NMR δ: 105.7 (C-8), 119.9 (C-4), 123.3 (C-7a), 125.6 (C-7), 129.0 (C-3' + C-5'), 130.0 (C-6), 131.3 (C-2' + C-6'), 131.6 (C-4'), 134.2 (C-1'), 134.7 (C-5), 140.3 (C-3a), 144.9 (C-3), 166.9 (C-1) ppm. ESI-MS: *m/z* 257.0291 [M+H]^+^; Anal. Calcd for C_15_H_9_ClO_2_: C, 70.19; H, 3.53. Found: C, 70.20; H, 3.49. 

#### 3.1.2. General Procedure for the Synthesis of Phthalazinones **1**–**4** and **12**

Benzalphthalides **B** (1 mol) were mixed with an excess of hydrazine hydrate (4 mL), and few drops of toluene, and the mixture maintained at 70–80 °C under stirring for 3–12 h. After cooling reaction mixtures were extracted with ethyl acetate and washed with water, dried over Na_2_SO_4_ and concentrated under reduced pressure to give crude products that were purified by flash chromatography on silica gel and/or crystallisation.

*4-(4-Chlorobenzyl)phthalazin-1(2H)-one* (**1**). Yield 77%. Colourless oil. IR (NaCl), ν_max_: 3159, 2902, 1664, 1609, 1488, 1258, 815, 798, 684 cm^−1^. ^1^H-NMR δ: 4.28 (s, 2H, H-9), 7.21 (d, *J* = 8.8 Hz, 2H, H-3' + H-5'), 7.27 (d, *J* = 8.8 Hz, 2H, H-2' + H-6'), 7.75 (m, 3H, H-5 + H-6 + H-7), 8.47 (dd, *J* = 7.5, 2.5 Hz, 1H, H-8), 11.74 (br s, 1H, NH) ppm. ^13^C-NMR δ: 38.2 (C-9), 125.2 (C-7), 127.1 (C-8), 128.3 (C-8a), 128.9 (C-4a + C-3' + C-5'), 129.9 (C-2' + C-6'), 131.5 (C-5); 132.7 (C-1'), 133.6 (C-6), 136.0 (C-4'), 146.0 (C-4), 160.6 (C-1) ppm. ESI-MS: *m/z* 271.0560 [M+H]^+^; Anal. Calcd for C_15_H_11_ClN_2_O: C, 66.55; H, 4.10; N, 10.35. Found: C, 66.49; H, 4.11; N, 10.30.

*4-(4-Methylsulfanylbenzyl)phthalazin-1(2H)-one* (**2**). Yield 50%. Colourless oil. IR (NaCl), ν_max_: 3188, 2920, 1657, 1492, 1260, 1017, 966, 793, 770 cm^−1^. ^1^H-NMR δ: 2.43 (*s*, 3H, SCH_3_), 4.26 (*s*, 2H, H-9), 7.18 (d, *J* = 8.0 Hz, 2H, H-3' + H-5'), 7.26 (d, *J* = 8.0 Hz, 2H, H-2' + H-6'), 7.73 (m, 3H, H-5 + H-6 + H-7), 8.46 (m, 1H, H-8), 11.48 (br s, 1H, NH) ppm. ^13^C-NMR δ: 15.7 (SCH_3_), 38.3 (C-9), 125.2 (C-7), 126.9 (C-8 + C-3' + C-5'), 128.2 (C-8a), 128.9 (C-2' + C-6'), 129.7 (C-4a), 131.3 (C-5); 133.4 (C-6), 134.4 (C-1'), 136.7 (C-4'), 146.2 (C-4), 160.8 (C-1) ppm. ESI-MS: *m/z* 283.0827 [M+H]^+^; Anal. Calcd for C_16_H_14_N_2_OS: C, 68.06; H, 5.00; N, 9.92. Found: C, 68.01; H, 4.96; N, 9.93.

*4-(3,4-Methylenedioxybenzyl)phthalazin-1(2H)-one* (**3**). Yield 100%. Colourless oil. IR (NaCl), ν_max_: 3,216, 2916, 2852, 1661, 1496, 1248, 925, 860, 764 cm^−1^. ^1^H-NMR δ: 4.20 (*s*, 2H, H-9), 5.90 (br s, 2H, OCH_2_O), 6.73 (br s, 1H, H-2'), 6.74 (br s, 2H, H-5' + H-6'), 7.74 (m, 3H, H-5 + H-6 + H-7), 8.47 (dd, *J* = 8.7, 2.0 Hz, 1H, H-8), 10.68 (br s, 1H, NH) ppm. ^13^C-NMR δ: 38.5 (C-9), 101.1 (OCH_2_O), 108.5 (C-5'), 108.9 (C-2'), 121.5 (C-6'), 125.4 (C-7), 127.0 (C-8), 128.3 (C-8a), 129.8 (C-4a), 131.3 (C-1'), 131.5 (C-5); 133.6 (C-6), 146.5 (C-4 + C-3'), 148.0 (C-4'), 160.2 (C-1). ESI-MS: *m/z* 281.0848 [M+H]^+^; Anal. Calcd for C_16_H_12_N_2_O_3_: C, 68.56; H, 4.32; N, 9.99. Found: C, 68.49; H, 4.26; N, 9.87.

*4-(3,4-Dimethoxybenzyl)phthalazin-1(2H)-one* (**4**). Yield 92%. Colourless oil. IR (NaCl), ν_max_: 3294, 2919, 1651, 1514, 1352, 1259, 1029, 860, 783, 730 cm^−1^. ^1^H-NMR δ: 3.82 (*s*, 6H, 2 × OCH_3_); 4.26 (s, 2H, H-9), 6.73 (br s, 1H, H-2'), 6.77 (d, *J* = 8.0 Hz, 1H, H-5'), 7.74 (m, 3H, H-5 + H-6 + H-7), 7.82 (dd, *J* = 8.0, 1.2 Hz, 1H, H-6'), 8.47 (m, 1H, H-8), 11.50 (*bs*, 1H, NH) ppm. ^13^C-NMR δ: 38.5 (C-9), 55.8 (2 × OCH_3_), 111.2 (C-5'), 115.5 (C-2'), 120.4 (C-6'), 125.4 (C-7), 126.9 (C-8), 128.2 (C-8a), 129.8 (C-4a), 130.1 (C-1'), 131.2 (C-5), 133.3 (C-6), 146.5 (C-4), 147.9 (C-4'), 149.1 (C-3'), 161.0 (C-1) ppm. ESI-MS: *m/z* 297.1161 [M+H]^+^; Anal. Calcd for C_17_H_16_N_2_O_3_: C, 68.91; H, 5.44; N, 9.45. Found: C, 68.88; H, 5.39; N, 9.46.

*1-Naphthylmethylphthalazin-1-one* (**12**). Yield 98%. Oil. IR (NaCl), ν_max_: 3417, 2919, 1653, 1595, 1470, 1023, 870, 787 cm^−1^. ^1^H-NMR δ: 4.18 (*s*, 2H, H-9), 7.12 (d, *J* = 7.0 Hz, 1H, H-4'), 7.53 (m, 1H, H-5'), 7.55 (m, 1H, H-9'), 7.57 (m, 1H, H-8'), 7.74 (m, 3H, H-5 + H-6 + H-7), 7.75 (m, 1H, H-10'), 7.76 (m, 1H, H-6'), 8.18 (dd, *J* = 8.2, 1.8 Hz, 1H, H-7'), 8.44 (dd, *J* = 8.4, 2.0 Hz, 1H, H-8), 11.00 (br s, 1H, NH) ppm. ^13^C-NMR δ: 35.3 (C-9), 123.1 (C-7'), 125.1 (C-7), 125.5 (C-5'), 126.1 (C-8'), 126.4 (C-9'), 127.2 (C-8 + C-4'), 127.7 (C-6'), 128.5 (C-8a), 128.9 (C-10'),129.8 (C-4a), 131.3 (C-5), 131.9 (C-2'), 133.3 (C-1' + C-3'), 133.4 (C-6), 146.3 (C-4), 160.7 (C-1) ppm. ESI-MS: *m/z* 287.1106 [M+H]^+^; Anal. Calcd for C_19_H_14_N_2_O: C, 79.70; H, 4.93; N, 9.78. Found: C, 79.71; H, 4.89; N, 9.72.

#### 3.1.3. General Procedure for the Synthesis of Phthalazinones **5**–**9** and **14**–**16**.

Benzalphthalides **B** (1 mol) were mixed with an excess of methylhydrazine (4 mL), the mixtures maintained at 70–80 °C under stirring for 4–11 h. After cooling reaction mixtures were extracted with ethyl acetate and washed with water, dried over Na_2_SO_4_ and concentrated under reduced pressure to give crude products that were purified by flash chromatography on silica gel. In the case of compounds **14**–**16** the starting benzalphthalides were 1:1 mixtures or regioisomers with the substituent at positions C-5 and C-6 and correspondingly yielded mixtures of 6(7)-substituted phthalazinones in the same proportion.

*4-(4-Chlorobenzyl)-2-methylphthalazin-1(2H)-one* (**5**). Yield 75%, oil. IR (NaCl): ν_max_ 3068, 2920, 1650, 1587, 1489, 1262, 1093, 815, 797, 749, 700 cm^−1^. ^1^H-NMR δ: 3.87 (s, 3H, CH_3_), 4.25 (s, 2H, H-9), 7.20 (d, *J* = 8.8 Hz, 2H, H-3' + H-5'), 7.26 (d, *J* = 8.8 Hz, 2H, H-2' + H-6'), 7.70 (m, 3H, H-5 + H-6 + H-7), 8.42 (m, 1H, H-8) ppm. ^13^C-NMR δ: 38.3 (C-9), 39.4 (CH_3_), 125.0 (C-7), 127.2 (C-8), 128.2 (C-8a), 128.9 (C-3' + C-5'), 129.2 (C-4a), 129.8 (C-2' + C-6'), 131.3 (C-5); 132.8 (C-1' + C-6), 136.4 (C-4'), 144.5 (C-4), 159.6 (C-1) ppm. ESI-MS: *m/z* 285.0716 [M+H]^+^; Anal. Calcd for C_16_H_13_ClN_2_O: C, 67.49; H, 4.60; N, 9.84. Found: C, 67.39; H, 4.52; N, 9.81.

*2-Methyl-4-(4-methylsulfanylbenzyl)phthalazin-1(2H)-one* (**6**). Yield 80%, Colourless oil. IR (NaCl): ν_max_ 2921, 2852, 1651, 1585, 1492, 1435, 1257, 1080, 810, 795, 775 cm^−1^. ^1^H-NMR δ: 2.43 (*s*, 3H, SCH_3_), 3.86 (s, 3H, NCH_3_), 4.23 (s, 2H, H-9), 7.17 (d, *J* = 8.8 Hz, 2H, H-3' + H-5'), 7.24 (d, *J* = 8.8 Hz, 2H, H-2' + H-6'), 7.67 (m, 3H, H-5 + H-6 + H-7), 8.43 (dd, *J* = 6.0, 2.9 Hz 1H, H-8) ppm. ^13^C-NMR δ: 15.9 (SCH_3_), 38.4 (C-9), 39.4 (NCH_3_),125.2 (C-7), 127.0 (C-8 + C-3' + C-5'), 128.2 (C-8a), 128.9 (C-2' + C-6'), 129.3 (C-4a), 131.2 (C-5); 132.8 (C-6), 134.8 (C-1'), 136.8 (C-4'), 143.9 (C-4), 159.6 (C-1) ppm. ESI-MS: *m/z* 297.0983 [M+H]^+^; Anal. Calcd for C _17_H_16_N_2_OS: C, 68.89; H, 5.44; N, 9.45. Found: C, 68.81; H, 5.43; N, 9.39; S, 10.76.

*2-Methyl-4-(3,4-methylenedioxybenzyl)phthalazin-1(2H)-one* (**7**). Yield 65%, Colourless oil. IR (NaCl): ν_max_ 2924, 2854, 1651, 1586, 1490, 1245, 1037, 925, 742, 698 cm^−1^. ^1^H-NMR δ: 3.89 (*s*, 3H, CH_3_), 4.19 (s, 2H, H-9), 5.90 (s, 2H, OCH_2_O), 6.70 (d, *J* = 8.0 Hz, 1H, H-5'), 6.71 (br s, 1H, H-2'), 6.74 (d, *J* = 8.0 Hz, 1H, H-6'), 7.69 (m, 3H, H-5 + H-6 + H-7), 8.41 (m, 1H, H-8) ppm. ^13^C-NMR δ: 38.6 (C-9), 39.4 (CH_3_), 100.9 (OCH_2_O), 108.3 (C-5'), 108.7 (C-2'), 121.3 (C-6'), 125.1 (C-7), 127.0 (C-8), 128.2 (C-8a), 129.2 (C-4a), 131.6 (C-1'), 131.1 (C-5); 132.6 (C-6), 145.1 (C-4), 146.3 (C-3'), 147.9 (C-4'), 159.6 (C-1) ppm. ESI-MS: *m/z* 295.1004 [M+H]^+^; Anal. Calcd for C_17_H_14_N_2_O_3_: C, 69.38; H, 4.79; N, 9.52. Found: C, 69.31; H, 4.77; N, 9.53.

*4-(3,4-Dimethoxybenzyl)-2-methylphthalazin-1(2H)-one* (**8**). Yield 93%, Colourless oil. IR (NaCl): ν_max_ 2926, 1652, 1515, 1453, 1260, 1236, 1029, 791, 744 cm^−1^. ^1^H-NMR δ: 3.83 (s, 6H, 2 × OCH_3_), 3.89 (*s*, 3H, CH_3_), 4.24 (s, 2H, H-9), 6.77 (d, *J* = 7.0 Hz, 1H, H-5'), 6.78 (s, 1H, H-2'), 6.79 (d, *J* = 7.0 Hz, 1H, H-6'), 7.69 (m, 3H, H-5 + H-6 + H-7), 8.44 (m, 1H, H-8) ppm. ^13^C-NMR δ: 38.6 (C-9), 39.4 (CH_3_), 55.9 (2 × OCH_3_), 111.3 (C-5'), 111.6 (C-2'), 120.5 (C-6'), 125.2 (C-7), 127.0 (C-8), 128.2 (C-8a), 128.4 (C-4a), 130.4 (C-1'), 131.2 (C-5), 132.7 (C-6), 145.2 (C-4), 147.9 (C-4'), 149.1 (C-3'), 159.6 (C-1) ppm. ESI-MS: *m/z* 311, 1317 [M+H]^+^; Anal. Calcd for C_18_H_18_N_2_O_3_: C, 69.66; H, 5.85; N, 9.03. Found: C, 69.59; H, 5.81; N, 9.04.

*4-(3,4,5-Trimethoxybenzyl)-2-methylphthalazin-1(2H)-one* (**9**). Yield 70%, oil. IR (NaCl): ν_max_: 2937, 2837, 1651, 1587, 1330, 804, 776, 743 cm^−1^. ^1^H-NMR 3.77 (s, 9H, 3 × OCH_3_), 3.89 (s, 3H, CH_3_), 4.21 (s, 2H, H-9), 6.44 (s, 2H, H-2' + H-6'), 7.68 (m, 3H, H-5 + H-6 + H-7), 8.42 (m, 1H, H-8) ppm. ^13^C-NMR δ: 39.2 (C-9), 39.4 (CH_3_), 56.1 (2 × OCH_3_), 60.8 (OCH_3_), 105.4 (C-2' + C-6'), 125.2 (C-7), 127.0 (C-8), 128.1 (C-8a), 129.4 (C-4a), 131.3 (C-5), 132.8 (C-6), 136.8 (C-1'), 145.0 (C-4), 153.4 (C-3' + C-4' + C-5'), 159.6 (C-1) ppm. ESI-MS: *m/z* 341.1423 [M+H]^+^; Anal. Calcd for C_19_H_20_N_2_O_4_: C, 67.05; H, 5.92; N, 8.23. Found: C, 67.01; H, 5.88; N, 8.24.

*2-Methyl-4-(naphthalen-2-ylmethyl)phthalazin-1(2H)-one* (**13**). Yield 99%, oil. IR (NaCl): ν_max_ 3052, 2926, 1651, 1584, 1257, 1033, 806, 785, 740, 691 cm^−1^. ^1^H-NMR δ: 3.91 (CH_3_) 4.39 (*s*, 2H, H-9), 7.38 (m, 1H, H-5), 7.40 (m, 1H, H-7), 7.52 (m, 1H, H-6), 7.60 (m, 3H, H-7' + H-8' + H-9'), 7.65 (m, 1H, H-6'), 7.75 (m, 1H, H-10'), 7.77 (br s, 1H, H-2'), 7.78 (m, 1H, H-5'), 8.46 (m, 1H, H-8) ppm. ^13^C-NMR δ: 39.5 (C-9), 39.8 (CH_3_), 125.6 (C-7), 126.1 (C-10'), 126.6 (C-7'), 127.0 (C-9'), 127.3 (C-8), 127.4 (C-6'), 128.0 (C-8'), 128.1 (C-8a), 128.5 (C-5'), 128.8 (C-2'), 129.7 (C-4a + C-5), 132.7 (C-1' + C-6), 133.9 (C-3'), 135.9 (C-4'), 145.3 (C-4), 160.0 (C-1) ppm. ESI-MS: *m/z* 301.1263 [M+H]^+^; Anal. Calcd for C_20_H_16_N_2_O: C, 79.98; H, 5.37; N, 9.33. Found: C, 79.91; H, 5.39; N, 9.30.

*4-(4-Chlorobenzyl)-2,6(7)-dimethylphthalazin-1(2H)-one* (**14**). Yield 93%, oil. IR (NaCl): ν_max_ 2922, 1651, 1618, 1490, 1091, 1015, 838 cm^−1^. ESI-MS: *m/z* 299.0873 [M+H]^+^; Anal. Calcd for C_17_H_15_ClN_2_O: C, 68.34; H, 5.06; N, 9.38. Found: C, 68.19; H, 4.95; N, 9.40.

*4-(4-Chlorobenzyl)-2,6-dimethylphthalazin-1(2H)-one* (**14a**). ^1^H-NMR δ: 2.42 (s, 3H, CH_3_), 3.84 (s, 3H, NCH_3_), 4.21 (s, 2H, H-9), 7.24–7.22 (m, 4H, H-2' + H-6' and H-3' + H-5'), 7.40 (br s, 1H, H-5), 7.52 (d, *J* = 8.4, 1H, H-7), 8.30 (d, *J* = 8.4, 1H, H-8) ppm. ^13^C-NMR δ: 21.8 (CH_3_), 37.7 (C-9), 39.1 (NCH_3_), 124.3 (C-5), 126.7 (C-8), 128.5 (C-3'+ C-5'), 128.6 (C-8a), 128.9 (C-4a), 129.5 (C-2' + C-6'), 132.2 (C-4'), 132.4 (C-7), 136.2 (C-1'), 144.1 (C-6), 143.3 (C-4), 159.2 (C-1) ppm. 

*4-(4-Chlorobenzyl)-2,7-dimethylphthalazin-1(2H)-one* (**14b**). ^1^H-NMR δ: 2.45 (s, 3H, CH_3_), 3.85 (s, 3H, NCH_3_), 4.20 (s, 2H, H-9), 7.24–7.22 (m, 4H, H-2' + H-6' and H-3' + H-5'), 7.45 (d, *J =* 8.4 Hz, 2H, H-5 + H-6), 8.30 (br s, 1H, H-8) ppm. ^13^C-NMR δ: 21.4 (CH_3_), 37.7 (C-9), 39.1 (NCH_3_), 124.7 (C-5), 126.4 (C-8), 126.6 (C-8a), 127.7 (C-4a), 128.5 (C-3'+ C-5'), 129.5 (C-2' + C-6'), 132.2 (C-4'), 136.2 (C-1'), 138.3 (C-6), 141.8 (C-7), 143.3 (C-4), 159.2 (C-1).

*4-(2,4-Dichlorobenzyl)-2,6(7)-dimethylphthalazin-1(2H)-one* (**15**). Yield 94%, oil. IR (NaCl): ν_max_ 2921, 1653, 1618, 1472, 1347, 1048, 860, 837 cm^−1^. ESI-MS: *m/z* 333, 0483 [M+H]^+^; Anal. Calcd for C_17_H_14_Cl_2_N_2_O: C, 61.28; H, 4.23; N, 8.41. Found: C, 61.30; H, 4.11; N, 8.30.

*4-(2,4-Dichlorobenzyl)-2,6-dimethylphthalazin-1(2H)-one* (**15a**). ^1^H-NMR δ: 2.46 (s, 3H, CH_3_), 3.81 (s, 3H, NCH_3_), 4.29 (s, 2H, H-9), 7.00 (d, *J* = 8.4 Hz, H-6'), 7.05 (dd, *J* = 8.4, 1.8 Hz, H-5'), 7.38 (d, *J* = 1.8 Hz, H-3'), 7.48 (s, 1H, H-5), 7.51 (d, *J* = 8.0 Hz, 1H, H-7), 8.32 (d, *J* = 8.0 Hz, 1H, H-8) ppm. ^13^C-NMR δ: 21.9 (CH_3_), 34.9 (C-9), 39.1 (NCH_3_), 124.0 (C-5), 126.7 (C-8), 127.0 (C-5'), 127.8 (C-8a), 129.1 (C-4a + C-6'), 130.7 (C-3'), 132.7 (C-7), 132.9 (C-2'), 134.1 (C-1' + C-4'), 143.1 (C-4), 143.6 (C-6), 159.3 (C-1) ppm. 

*4-(2,4-Dichlorobenzyl)-2,7-dimethylphthalazin-1(2H)-one* (**15b**). ^1^H-NMR δ: 2.49 (s, 3H, CH_3_), 3.82 (s, 3H, NCH_3_), 4.29 (s, 2H, H-9), 6.97 (d, *J* = 8.4 Hz, H-6'), 7.10 (dd, *J* = 8.4, 1.8 Hz, H-5'), 7.38 (d, *J* = 1.8 Hz, H-3'), 7.38 (d, 1H, *J* = 7.7 Hz, H-6), 7.50 (d, 1H, *J* = 7.7 Hz, H-5), 8.32 (br s, 1H, H-8) ppm. ^13^C-NMR δ: 21.6 (CH_3_), 34.9 (C-9), 39.1 (NCH_3_), 124.4 (C-5), 126.6 (C-8), 127.0 (C-5'), 125.6 (C-8a), 129.1 (C-4a + C-6'), 130.7 (C-3'), 142.1 (C-7), 132.9 (C-2'), 134.1 (C-1' + C-4'), 143.4 (C-4), 134.1 (C-6), 159.3 (C-1) ppm. 

*4-(3,4-Dichlorobenzyl)-2,6(7)-dimethylphthalazin-1(2H)-one* (**16**). Yield 86%, oil. IR (NaCl): ν_max_ 2921, 1651, 1618, 1470, 1347, 1031, 823 cm^−1^. ESI-MS: *m/z* 333.0483 [M+H]^+^; Anal. Calcd for C_17_H_14_Cl_2_N_2_O_2_: C, 61.28; H, 4.23; N, 8.41. Found: C, 61.17; H, 4.12; N, 8.49.

*4-(3,4-Dichlorobenzyl)-2,6-dimethylphthalazin-1(2H)-one* (**16a**). ^1^H-NMR δ: 2.45 (s, 3H, CH_3_), 3.84 (s, 3H, NCH_3_), 4.20 (s, 2H, H-9), 7.10 (dd, *J* = 8.6, 2.0 Hz, H-6'), 7.33 (d, *J* = 8.6 Hz, H-5'), 7.35 (d, *J* = 2.0 Hz, H-2'), 7.39 (s, 1H, H-5), 7.51 (d, *J* = 8.0 Hz, 1H, H-7), 8.32 (d, *J* = 9.0 Hz, 1H, H-8) ppm. ^13^C-NMR δ: 22.0 (CH_3_), 37.6 (C-9), 39.2 (NCH_3_), 124.3 (C-5), 125.8 (C-8a), 127.1 (C-8), 128.0 (C-4a + 5'), 130.2 (C-2'), 130.4 (C-6'), 130.7 (C-3'), 132.5 (C-4'), 132.8 (C-7), 138.1 (C-1'), 143.5 (C-4), 143.6 (C-6), 159.4 (C-1) ppm.

*4-(3,4-Dichlorobenzyl)-2,7-dimethylphthalazin-1(2H)-one* (**16b**). ^1^H-NMR δ: 2.48 (s, 3H, CH_3_), 3.86 (s, 3H, NCH_3_), 4.20 (s, 2H, H-9), 7.11 (dd, *J* = 8.0, 2.0 Hz, H-6'), 7.32 (d, *J* = 8.0, 1.8 Hz, H-5'), 7.35 (d, *J* = 2.0 Hz, H-2'), 7.48 (dd, 1H, *J* = 7.7,1.5 Hz, H-6), 7.50 (d, 1H, *J* = 7.7 Hz, H-5), 8.23 (br s, 1H, H-8) ppm. ^13^C-NMR δ: 21.6 (CH_3_), 37.8 (C-9), 39.2 (NCH_3_), 124.6 (C-5), 125.8 (C-8a), 126.8 (C-8), 128.0 (C-5'), 129.1 (C-4a), 130.4 (C-6'), 130.7 (C-3'), 130.2 (C-2'), 132.5 (C-4'), 134.1 (C-6), 138.1 (C-1'), 142.2 (C-7), 143.8 (C-4), 159.4 (C-1) ppm.

#### 3.1.4. Procedure for the Synthesis of Compounds **10** and **11**

A mixture of phthalazinone **1** (0.20 mmol), ethyl bromide or allyl bromide (0.22 mmol), potassium carbonate (33 mg) and acetonitrile (5 mL) were maintained under reflux for 25 h. Solvent was removed under vacuum and the crude mixture dissolved in ethyl acetate, washed with water, dried over Na_2_SO_4_ and concentrated under reduced pressure to give crude products that were purified by flash chromatography on silica gel.

*4-(4-Chlorobenzyl)-2-ethylphthalazin-1(2H)-one* (**10**). Yield 89%, Colourless oil. IR (NaCl): ν_max_ 2930, 1650, 1585, 1350, 1262, 1090, 830, 798, 691 cm^−1^. ^1^H-NMR δ: 1.43 (t, *J* = 7.3 Hz, 3H, CH_3_), 4.26 (*s*, 2H, H-9), 4.33 (q, *J* = 7.3 Hz, 2H, CH_2_), 7.18 (d. *J* = 8.5 Hz, H-3' + H-5'), 7.26 (d, *J* = 8.5 Hz, 2H, H-2' + H-6'), 7.66 (m, 3H, H-5 + H-6 + H-7), 8.45 (m, 1H, H-8) ppm. ^13^C-NMR δ: 13.6 (CH_3_), 38.3 (C-9), 46.2 (CH_2_), 124.9 (C-7), 127.3 (C-8), 128.4 (C-8a), 128.8 (C-3' + C-5'), 129.0 (C-4a), 129.7 (C-2' + C-6'), 131.2 (C-5); 132.7 (C-1'), 132.8 (C-6), 136.5 (C-4'), 144.6 (C-4), 159.0 (C-1) ppm. ESI-MS: *m/z* 299.0873 [M+H]^+^; Anal. Calcd for C_17_H_15_ClN_2_O: C, 68.34; H, 5.06; N, 9.38. Found: C, 68.27; H, 5.04; N, 9.30.

*2-Allyl-4-(4-Chlorobenzyl)phthalazin-1(2H)-one* (**11**). Yield 73%, oil. IR (NaCl): ν_max_ 3073, 2930, 1655, 1586, 1490, 1092, 810, 796 cm^−1^. ^1^H-NMR δ: 4.25 (*s*, 2H, H-9), 4.85 (m, 2H, CH_2_), 5.20/5.27 (m, 2H, =CH_2_), 6.06 (m, 1H, CH=), 7.18 (d. *J* = 8.2 Hz, H-3' + H-5'), 7.22 (d, *J* = 8.2 Hz, 2H, H-2' + H-6'), 7.67 (m, 3H, H-5 + H-6 + H-7), 8.42 (m, 1H, H-8) ppm. ^13^C-NMR δ: 38.2 (C-9), 53.4 (CH_2_), 117.8 (=CH_2_), 124.8 (C-7), 127.3 (C-8), 128.3 (C-8a), 128.7 (C-3' + C-5'), 129.1 (C-4a), 129.6 (C-2' + C-6'), 130.1 (C-1'), 131.2 (C-5); 132.5 (CH=), 132.8 (C-6), 136.2 (C-4'), 144.8 (C-4), 158.9 (C-1) ppm. ESI-MS: *m/z* 311.0873 [M+H]^+^; Anal. Calcd for C_18_H_15_ClN_2_O: C, 69.57; H, 4.86; N, 9.01. Found: C, 69.48; H, 4.80; N, 9.02.

#### 3.1.5. General Procedure for the Synthesis of Phthalazinones **17**–**22**

A solution of the corresponding benzalphthalide **B** (1 mol), methylhydrazine (3 mL) in dichloromethane (6 mL) was absorbed in silica gel (10:1 respecting the benzalphthalide). The solvent was removed under vacuum and the mixture MW irradiated (350 W) for 1–6 minutes. Then, 3 drops of water were added and stirred for 20 min at room temperature. Ethyl acetate was added to the mixture and the silica gel filtered out. The solvent was removed under vacuum and the crude mixture purified by flash chromatography on silica gel. Phthalazinones **17**–**19** were obtained as 1:1 mixtures of regioisomers at the 6/7 positions. 

*4-(4-Chlorobenzyl)-6(7)-hydroxycarbonyl-2-methylphthalazin-1(2H)-one* (**17**). Yield 90%, oil. IR (NaCl): ν_max_ 3430–2715, 1720, 1645, 1614, 1352, 1088, 803, 720 cm^−1^. ESI-MS: *m/z* 330.0611 [M+H]^+^; Anal. Calcd for C_17_H_13_ClN_2_O_3_: C, 62.11; H, 3.99; N, 8.52. Found: C, 62.15; H, 3.90; N, 8.50.

*4-(4-Chlorobenzyl)-6-hydroxycarbonyl-2-methylphthalazin-1(2H)-one* (**17a**). ^1^H-NMR (CD_3_OD + CDCl_3_) δ: 3.88 (s, 3H, NCH_3_), 4.29 (*s*, 2H, H-9), 7.18–7.30 (m, 4H, H-2' + H-6' and H-3'+ H-5'), 8.45 (br s, 1H, H-5), 8.33 (d, *J* = 8.4 Hz, 1H, H-7), 7.73 (d, *J* = 8.4 Hz, 1H, H-8) ppm. ^13^C-NMR (CD_3_OD + CDCl_3_) δ: 38.0 (C-9), 39.4 (NCH_3_), 125.3 (C-8), 127.7 (C-8a), 128.7 (C-3' + H-5'), 128.9 (C-5), 129.7 (C-2' + H-6'), 130.2 (C-4a), 131.6 (C-4'), 132.6 (C-6), 133.4 (C-7), 135.7 (C-1'), 144.8 (C-4), 159.5 (C-1), 166.7 (COOH) ppm.

*4-(4-Chlorobenzyl)-7-hydroxycarbonyl-2-methylphthalazin-1(2H)-one* (**17b**). ^1^H-NMR (CD_3_OD + CDCl_3_) δ: 3.88 (s, 3H, NCH_3_), 4.31 (s, 2H, H-9), 7.18–7.30 (m, H-2' + H-6' and H-3' + H-5'), 8.33 (d, *J* = 8.0 Hz, 1H, H-5), 8.49 (d, *J* = 8.0 Hz, 1H, H-6), 9.09 (s, 1H, H-8) ppm. ^13^C-NMR (CD_3_OD + CDCl_3_) δ: 37.8 (C-9), 39.4 (NCH_3_), 127.0 (C-8), 127.2 (C-5), 127.7 (C-8a), 128.7 (C-3' + H-5'), 129.7 (C-2' + H-6'), 130.3 (C-4a), 131.6 (C-6), 131.7 (C-4'), 135.7 (C-1'), 145.5 (C-4), 134.8 (C-7), 159.2 (C-1), 166.5 (COOH) ppm.

*4-(4-Chlorobenzyl)-6(7)-hydroxymethyl-2-methylphthalazin-1(2H)-one* (**18**). Yield 91%, Colourless oil. IR (NaCl): ν_max_ 3306, 1632, 1617, 1582, 1356, 1060, 844, 821 cm^−1^. ESI-MS: *m/z* 313.0611 [M+H]^+^; Anal. Calcd for C_17_H_15_ClN_2_O_3_: C, 64.87; H, 4.80; N, 8.90. Found: C, 64.76; H, 4.70; N, 8.87.

*4-(4-Chlorobenzyl)-6-hydroxymethyl-2-methylphthalazin-1(2H)-one* (**18a**). ^1^H-NMR δ: 3.84 (s, 3H, NCH_3_), 4.22 (s, 2H, H-9), 4.80 (s, 2H, CH_2_OH), 7.16–7.22 (m, 4H, H-2'+ H-6'and H-3' + H-5'), 7.60 (d, *J* = 8.0 Hz, 1H, H-7), 7.66 (s, 1H, H-5), 8.31 (d, *J* = 8.0 Hz, 1H, H-8) ppm. ^13^C-NMR δ: 38.1 (C-9), 39.5 (NCH_3_), 64.3 (CH_2_), 122.1 (C-5), 127.2 (C-8a), 127.3 (C-8), 128.9 (C-7 + C-3' + H-5'), 129.2 (C-4a), 129.8 (C-2' + H-6'), 132.6 (C-4'), 136.3 (C-1'), 144.8 (C-4), 146.6 (C-6), 159.6 (C-1) ppm.

*4-(4-Chlorobenzyl)-7-hydroxymethyl-2-methylphthalazin-1(2H)-one* (**18b**). ^1^H-NMR δ: 3.84 (s, 3H, NCH_3_), 4.23 (s, 2H, H-9), 4.82 (s, 2H, CH_2_OH), 7.16–7.22 (m, 4H, H-2' + H-6' and H-3' + H-5'), 7.64 (d, *J* = 8.4 Hz, 1H, H-5), 7.72 (dd, *J* = 8.4, 1.5 Hz, 1H, H-6), 8.36 (br s, 1H, H-8) ppm. ^13^C-NMR δ: 38.3 (C-9), 39.5 (NCH_3_), 64.3 (CH_2_), 124.5 (C-5), 125.3 (C-8), 127.2 (C-8a), 128.3 (C-4a), 128.9 (C-3' + H-5'), 129.8 (C-2' + H-6'), 131.5 (C-6), 132.6 (C-4'), 136.3 (C-1), 144.6 (C-4), 145.2 (C-7), 159.6 (C-1) ppm.

*4-(4-Chlorobenzyl)-6(7)-nitro-2-methylphthalazin-1(2H)-one* (**19**). Yield 53%, yellowish oil. IR (NaCl): ν_max_ 2918, 1662, 1618, 1531, 1344, 1090, 794 cm^−1^. ESI-MS: *m/z* 329.0567 [M+H]^+^; Anal. Calcd for C_16_H_12_ClN_3_O_3_: C, 58.28; H, 3.67; N, 12.74. Found: C, 58.18; H, 3.72; N, 12.50.

*4-(4-Chlorobenzyl)-6-nitro-2-methylphthalazin-1(2H)-one* (**19a**). ^1^H-NMR (400 MHz) δ: 3.82 (*s*, 3H, CH_3_), 4.23 (*s*, 2H, H-9), 7.08–7.18 (m, 4H, H-3' + H-5' + H-2' + H-6'), 8.47 (*s*, 1H, H-5), 7.72 (d, *J* = 8.7 Hz, 1H, H-7), 8.53 (d, *J* = 8.7Hz, 1H, H-8) ppm. ^13^C-NMR (100 MHz) δ: 38.5 (C-9), 39.8 (CH_3_), 123.3 (C-8), 125.1 (C-5); 129.0 (C-8a), 129.3 (C-3' + C-5'), 129.8 (C-7 + C-2' + C-6'), 131.9 (C-4'), 132.8 (C-4a), 135.3 (C-1'), 143.4 (C-4), 149.0 (C-6), 158.4 (C-1) ppm. 

*4-(4-Chlorobenzyl)-7-nitro-2-methylphthalazin-1(2H)-one* (**19b**). ^1^H-NMR (400 MHz) δ: 3.82 (*s*, 3H, CH_3_), 4.23 (*s*, 2H, H-9), 7.08–7.18 (m, 4H, H-3' + H-5' + H-2'+ H-6'), 8.37 (d, *J* = 8.4 Hz, 1H, H-6), 8.49 (d, *J* = 8.4 Hz, 1H, H-5), 9.18 (*s*, 1H, H-8) ppm. ^13^C-NMR (100 MHz) δ: 38.3 (C-9), 39.8 (CH_3_), 120.7 (C-8), 126.9 (C-6), 129.0 (C-8a), 129.3 (C-3' + C-5'), 129.8 (C-5 + C-2' + C-6'), 131.9 (C-4'), 133.8 (C-4a), 135.5 (C-1'), 143.1 (C-4), 150.2 (C-7), 158.1 (C-1) ppm. 

*6,7-Dichloro-4-(4-chlorobenzyl)-phthalazin-1(2H)-one* (**20**). Yield 64%, Colourless oil. IR (NaCl): ν_max_ 2943, 1652, 1490, 1090, 1015, 804, 732 cm^−1^. ^1^H-NMR δ: 3.85 (s, 3H, CH_3_), 4.20 (s, 2H, H-9), 7.18 (d, *J* = 8.4, 2H, H-2' + H-6'), 7.28 (d, *J* = 8.4 Hz, 2H, H-3' + H-5'), 7.71 (s, 1H, H-5), 8.49 (s, 1H, H-8) ppm. ^13^C-NMR δ: 38.1 (C-9), 39.6 (CH_3_), 126.7 (C-8), 127.5 (C-8a), 128.4 (C-1'), 129.1 (C-5 + C-3' + C-5'), 129.7 (C-2' + C-6'), 133.0 (C-4'), 135.5 (C-7), 136.5 (C-4a), 138.0 (C-6), 143.0 (C-4), 157.9 (C-1) ppm. ESI-MS: *m/z* 352.9937 [M+H]^+^; Anal. Calcd for C_16_H_11_Cl_3_N_2_O: C, 54.34; H, 3.14; N, 7.92. Found: C, 54.40; H, 3.07; N, 7.79.

*6,7-Dichloro-4-(2,4-dichlorobenzyl)-phthalazin-1(2H)-one* (**21**). Yield 86%, oil. IR (NaCl): ν_max_ 2923, 1660, 1581, 1471, 1128, 1101, 850 cm^−1^. ^1^H-NMR (400 MHz) 3.80 (s, 3H, CH_3_), 4.20 (s, 2H, H-9), 7.03 (d, *J* = 8.0 Hz, 1H, H-6'), 7.15 (dd, *J* = 8.0, 1.7 Hz, 1H, H-5'), 7.47 (d, *J* = 1.7 Hz, 1H, H-3'), 7.76 (s, 1H, H-5), 8.53 (s, 1H, H-8) ppm. ^13^C-NMR δ: 35.0 (C-9), 39.5 (CH_3_), 126.3 (C-8), 127.3 (C-8a), 127.4 (C-5'), 128.4 (C-1'), 129.1 (C-5), 129.5 (C-3'), 131.0 (C-6'), 133.3 (C-2'), 133.6 (C-4'), 134.3 (C-7), 136.6 (C-4a), 138.2 (C-6), 142.0 (C-4), 158.0 (C-1) ppm. ESI-MS: *m/z* 386.9547 [M+H]^+^; Anal. Calcd for C_16_H_10_Cl_4_N_2_O: C, 49.52; H, 2.60; N, 7.22. Found: C, 49.43; H, 2.71; N, 7.14.

*6,7-Dichloro-4-(3,4-dichlorobenzyl)-phthalazin-1(2H)-one* (**22**). Yield 89%, oil. IR (NaCl): ν_max_ 2921, 1651, 1618, 1470, 1347, 1031, 823 cm^−1^. ^1^H-NMR δ: 3.81 (s, 3H, CH_3_), 4.25 (s, 2H, H-9), 7.09 (d, *J* = 8.6 Hz, 1H, H-6'), 7.10 (d, *J* = 8.6 Hz, 1H, H-5'), 7.40 (br s, 1H, H-2'), 7.80 (s, 1H, H-5), 8.49 (s, 1H, H-8) ppm. ^13^C-NMR δ: 37.8 (C-9), 39.6 (CH_3_), 126.1 (C-8), 127.3 (C-8a), 128.1 (C-5'), 129.0 (C-5), 130.8 (C-6'), 131.9 (C-2'), 132.3 (C-3'), 133.5 (C-4'), 133.9 (C-7), 136.3 (C-4a), 137.5 (C-1'), 138.3 (C-6), 143.1 (C-4), 158.2 (C-1) ppm. ESI-MS: *m/z* 386.9547 [M+H]^+^; Anal. Calcd for C_16_H_10_Cl_4_N_2_O: C, 49.52; H, 2.60; N, 7.22. Found: C, 49.61; H, 2.53; Cl, 36.57; N, 7.17.

#### 3.1.6. Synthesis of Phthalazinone Carboxymethyl ester **23**

The phthalazinone **17** (20 mg, 0,06 mmoles) was treated with a saturated solution diazomethane in ether (2 mL), and maintaind in darkness at room temperature overnight. The solvent was removed to give 22 mg (99%) of the ester **23**, as a regioisomeric mixture. 

*4-(4-Chlorobenzyl)-6(7)-methoxycarbonyl-2-methylphthalazin-1(2H)-one* (**23**). Oil. IR (NaCl): ν_max_ 2928, 1704, 1652, 1614, 1490, 1347, 1090, 1015, 845 cm^−1^. ESI-MS: *m/z* 343.0771 [M+H]^+^; Anal. Calcd for C_18_H_15_ClN_2_O_3_: C, 63.07; H, 4.41; N, 8.17. Found: C, 62.97; H, 4.51; N, 8.22.

*4-(4-Chlorobenzyl)-6-methoxycarbonyl-2-methylphthalazin-1(2H)-one* (**23a**). ^1^H-NMR δ: 3.88 (*s,* 3H, CH_3_), 3.97 (s, 3H, OCH_3_), 4.28 (s, 2H, H-9), 7.19 (d, *J* = 8.8 Hz, 2H, H-3' + H-5'), 7.27 (d, *J* = 8.8 Hz, 2H, H-2' + H-6'), 7.70 (d, *J* = 8.8 Hz, 1H, H-7), 8.29 (d, *J* = 8.8 Hz, 1H, H-8), 8.40 (s, 1H, H-5) ppm. ^13^C-NMR δ: 38.2 (C-9), 39.6 (CH_3_), 52.7 (OCH_3_), 125.4 (C-5); 128.2 (C-8a), 129.0 (C-8 + C-3' + C-5'), 129.8 (C-2' + C-6'), 131.3 (C-7), 132.0 (C-4'), 133.0 (C-4a), 133.1 (C-6), 136.0 (C-1'), 144.1 (C-4), 159.1 (C-1), 165.6 (COO) ppm.

*4-(4-Chlorobenzyl)-7-methoxycarbonyl-2-methylphthalazin-1(2H)-one* (**23b**). ^1^H-NMR δ: 3.88 (s, 3H, CH_3_), 3.97 (s, 3H, OCH_3_), 4.30 (s, 2H, H-9), 7.19 (d, *J* = 8.8 Hz, 2H, H-3' + H-5'), 7.27 (d, *J* = 8.8 Hz, 2H, H-2' + H-6'), 8.29 (d, *J* = 8.4 Hz, 1H, H-6), 8.51 (d, *J* = 8.4 Hz, 1H, H-5), 9.03 (s, 1H, H-8). ^13^C-NMR δ: 38.4 (C-9), 39.6 (CH_3_), 52.7 (OCH_3_), 126.9 (C-5); 127.8 (C-8), 128.2 (C-8a), 129.0 (C-3' + C-5'), 129.8 (C-2' + C-6'), 132.0 (C-4'), 132.6 (C-4a), 132.9 (C-6), 134.0 (C-7), 136.0 (C-1'), 144.8 (C-4), 159.1 (C-1), 165.6 (COO).

#### 3.1.7. Synthesis of the Phthalazinone Aldehyde **24**

To a three-neck round-bottom flask filled with dichloromethane (15 mL) and a stirring bar, two compensated pressure addition funnels were adapted. Air was removed, the system filled with Ar and taken to −55 °C, then a solution of 2M oxallyl chloride in dichloromethane (1.10 mL, 2.20 mmol) was added. Five min later a mixture of dimethylsulfoxide (0.4 mL, 4.44 mmol) in dichloromethane (2.3 mL) was added dropwise. After 5 min a solution of phthalazinone **18** (230 mg, 0.73 mmol) in dichloromethane (6.5 mL) was added slowly. The mixture was maintained with stirring for 30 min at −55 °C. Then, triethylamine (1.0 mL, 7.20 mmol) was added and the mixture taken to 0 °C for 60 min. Then, water (5 mL) was added to the mixture, which was transferred to a separatory funnel, where it was washed with aqueous solutions of 2N HCl, NaHCO_3_ (saturated) and NaCl to pH = 7. The organic layer was dried over Na_2_SO_4_, concentrated under reduced pressure to give a crude mixture, that was purified by flash chromatography on silica gel in CH_2_Cl_2_/AcOEt (9:1) to provide 138 mg (61%) of aldehyde **24**.

*4-(4-Chlorobenzyl)-6(7)-formyl-2-methylphthalazin-1(2H)-one* (**24**). Oil. IR (NaCl): ν_max_ 2928, 1704, 1652, 1614, 1490, 1347, 1090, 1015, 845 cm^−1^. ESI-MS: *m/z* 313.0666 [M+H]^+^; Anal. Calcd. for C_17_H_13_ClN_2_O_2_: C, 65.29; H, 4.19; N, 8.96. Found: C, 65.31; H, 4.12; N, 8.83.

*4-(4-Chlorobenzyl)-6-formyl-2-methylphthalazin-1(2H)-one* (**24a**). ^1^H-NMR δ: 3.89 (s, 3H, CH_3_), 4.32 (s, 2H, H-9), 7.19 (d, *J* = 8.8 Hz, 2H, H-3' + H-5'), 7.29 (d, *J* = 8.8 Hz, 2H, H-2' + H-6'), 8.17 (s, 1H, H-5), 8.18 (d, *J* = 8.8 Hz, 1H, H-7), 8.61 (d, *J* = 8.8 Hz, 1H, H-8), 10.10 (s, 1H, CHO) ppm. ^13^C-NMR δ: 38.3 (C-9), 39.7 (CH_3_), 127.2 (C-5); 128.8 (C-8a), 128.6 (C-8), 129.1 (C-3' + C-5'), 129.8 (C-2 '+ C-6'), 130.7 (C-7), 131.9 (C-4a), 133.0 (C-4'), 135.8 (C-1'), 138.9 (C-6), 144.8 (C-4), 158.8 (C-1), 190.8 (CHO) ppm.

*4-(4-Chlorobenzyl)-7-formyl-2-methylphthalazin-1(2H)-one* (**24b**). ^1^H-NMR δ: 3.90 (s, 3H, CH_3_), 4.29 (s, 2H, H-9), 7.19 (d, *J* = 8.8 Hz, 2H, H-3'+ H-5'), 7.29 (d, *J* = 8.8 Hz, 2H, H-2'+ H-6'), 7.77 (d, *J* = 8.4 Hz, 1H, H-6), 8.17 (d, *J* = 8.4 Hz, 1H, H-5), 8.90 (br s, 1H, H-8), 10.17 (s, 1H, CHO) ppm. ^13^C-NMR δ: 38.3 (C-9), 39.7 (CH_3_), 126.1 (C-5); 128.8 (C-8a), 129.1 (C-3'+C-5'), 129.6 (C-8), 129.8 (C-2' + C-6'), 131.3 (C-6), 133.0 (C-4'), 135.8 (C-1'), 137.8 (C-4a), 138.9 (C-7), 144.1 (C-4), 159.0 (C-1), 190.7 (CHO) ppm.

#### 3.1.8. Synthesis of the 6(7)hydroxylimino-phthalazinone **25**

To a solution of **24** (100 mg, 0.32 mmol) in ethanol (5 mL), dry pyridine (83 μL, 1.03 mmol) and hydroxylamine clorhydrate (25 mg, 0.35 mmol) were added. The mixture was refluxed under stirring for 2 hours. Solvents were removed under vacuum and the mixture dissolved in ethyl acetate. The organic layer was washed with solutions of 2N HCl and NaCl to pH = 7, dried over Na_2_SO_4_, and taken do dryness to give 95 mg (92%) of the regioisomers **25**.

*4-(4-Chlorobenzyl)-6(7)-hydroxylimino-2-methylphthalazin-1(2H)-one* (**25**). Oil. IR (NaCl): ν_max_ 3441, 2927, 1632, 1579, 1111, 995, 796, 674 cm^−1^. ESI-MS: *m/z* 328.0775 [M+H]^+^; Anal. Calcd for C_17_H_14_ClN_3_O_2_: C, 62.30; H, 4.31; N, 12.82. Found: C, 62.35; H, 4.38; N, 12.83.

*4-(4-Chlorobenzyl)-6-hydroxylimino-2-methylphthalazin-1(2H)-one* (**25a**). ^1^H-NMR (400 MHz, DMSO-d6) δ: 3.72 (s, 3H, CH_3_), 4.29 (s, 2H, H-9), 7.33-7.34 (m, 4H, H-2' + H-6' and H-3' + H-5'), 7.90 (d, *J* = 8.5 Hz, 1H, H-8), 8.04 (d, *J* = 8.5 Hz, 1H, H-7), 8.33 (s, 1H, HC=N), 8.41 (s, 1H, H-5) ppm. ^13^C-NMR (100 MHz) δ: 36.8 (C-9), 39.1 (CH_3_), 124.3 (C-5); 126.2 (C-8), 127.8 (C-4a), 128.5 (C-3' + C-5'), 128.7 (C-8a), 130.0 (C-7), 130.3 (C-2' + C-6'), 131.2 (C-4'), 136.2 (C-6), 144.3 (C-4), 137.0 (C-1'), 147.1 (HC=N) 158.2 (C-1), ppm.

*4-(4-Chlorobenzyl)-7-hydroxylimino-2-methylphthalazin-1(2H)-one* (**25b**). ^1^H-NMR (400 MHz, MDSO-d6) δ: 3.71 (s, 3H, CH_3_), 4.29 (s, 2H, H-9), 7.33-7.34 (m, 4H, H-2' + H-6' and H-3' + H-5'), 8.04 (d, *J* = 8.4 Hz, 1H, H-6), 8.33 (s, 1H, HC=N), 8.50 (d, *J* = 8.4 Hz, 1H, H-5), 8.60 (d, *J* = 8.5 Hz, 1H, H-8 ppm. ^13^C-NMR (100 MHz) δ: 38.8 (C-9), 39.1 (CH_3_), 123.9 (C-5); 127.6 (C-8), 127.9 (C-8a), 128.0 (C-4a), 128.5 (C-3' + C-5'), 130.3 (C-6 + C-2'+C-6'), 131.2 (C-4'), 137.0 (C-7 + C-1'), 144.3 (C-4), 147.1 (HC=N) 158.2 (C-1) ppm.

### 3.2. Antifungal Evaluation

#### 3.2.1. Microorganisms And Media

For the antifungal evaluation, standardized strains from the American Type Culture Collection (ATCC), Manassas, Virginia, USA, and Culture Collection of the *Reference Center of Mycology* (CCC), Faculty of Biochemical and Pharmacuetical Sciences, Suipacha 531-(2000)-Rosario, Argentina were used in a first instance of screening: *C. albicans* ATCC 10231, *S. cerevisiae* ATCC 9763, *C. neoformans* ATCC 32264, *A. flavus* ATCC 9170, *A. fumigatus* ATTC 26934, *A. niger* ATCC 9029, *T. rubrum* CCC 110, *T. mentagrophytes* ATCC 9972, *M. gypseum* CCC 115, *M. canis* CCC 113 and *E. floccosum* CCC 112.

Active compounds were tested against clinical isolates from the Malbrán Institute [(MI), Av. Velez Sarsfield 563. Buenos Aires)]. The isolates included eight strains of *C. neoformans.* The voucher specimen numbers are presented in Table 3. Strains were grown on Sabouraud-chloramphenicol agar slants for 48 h at 30 °C, maintained on slopes of Sabouraud-dextrose agar (SDA, Oxoid, Hampshire, UK) and sub-cultured every 15 d to prevent pleomorphic transformations. Inocula of cell or spore suspensions were obtained according to reported procedures and adjusted to 1-5 x10^3^ cells/spores with colony forming units (CFU) per mL [18,19].

#### 3.2.2. Antifungal Susceptibility Testing

Minimum Inhibitory Concentration (MIC) of each compound was determined by using broth microdilution techniques according to the guidelines of the Clinical and Laboratory Standards Institute (CLSI, formerly National Committee for Clinical Laboratory Standards, NCCLS) for yeasts (M27-A3) and for filamentous fungi (M 38 A2) [18,19].

MIC values were determined in RPMI-1640 (Sigma, St. Louis, MO, USA) buffered to pH 7.0 with MOPS. Microtiter trays were incubated at 35 °C for yeasts and at 28–30 °C for the rest of fungi in a moist, dark chamber, and MICs were visually recorded at 48 h for yeasts, and at a time according to the control fungus growth, for the rest of fungi. 

For the assay, stock solutions of pure compounds were two-fold diluted with RPMI from 250–0.98 μg/mL (final volume = 100 μL) and a final DMSO concentration ≤ 1%. A volume of 100 μL of inoculum suspension was added to each well with the exception of the sterility control where sterile water was added to the well instead. Terbinafine, amphotericin B, voriconazole and itraconazole, were used as positive controls.

Endpoints were defined as the lowest concentration of drug resulting in total inhibition (MIC_100_) of visual growth compared to the growth in the control wells containing no antifungal. MIC_80_ and MIC_50_ were defined as the lowest concentration of a compound that induced 80% or 50% reduction of the growth control respectively (culture media with the microorganism but without the addition of any compound) and was determined spectrophotometrically with the aid of a VERSA Max microplate reader (Molecular Devices, Sunnyvale, CA, USA).

### 3.3. Computational Methods

All calculations were carried out using the Gaussian 03 program [25]. The search for low-energy conformations on the potential energy surface for compound **5** was carried out by first using semi-empirical PM6 calculations. Subsequently, DFT (B3LYP/6-31G (d,p)) calculations were used in the geometry optimisation jobs. Minima were characterized through harmonic frequency analysis. Correlations effects were included using Density Functional Theory (DFT) with the Becke-3-Lee-Yang-parr (RB3LYP) [26] functional and 6-31++G(d,p) basis set for all complexes obtained at the lower level of computation. During the DFT calculations, the RHF/6-31G geometries were kept fixed.

Potential energy curves (PEC) have been obtained via one-dimensional (1D)-scans using DFT (B3LYP/6-31G (d,p)) calculations. In these curves the energy has been calculated at 30 ° intervals of the dihedral angles.

The electronic study of the compounds was carried out by using molecular electrostatic potentials. MEPs have been shown to provide reliable information, both on the interaction sites of the molecules with point charges and on the comparative reactivities of these sites [27]. These MEPs were calculated using B3LYP/6-311++G(d,p) single point calculations from the MOLEKEL program [28].

## 4. Conclusions

In summary, we have described here a group of 2-methylphthalazin-1(*2H*)-one derivatives acting as antifungal agents. Among them, the compound 4-(4-chlorobenzyl)-2-methylphthalazin-1(*2H*)-one (**5**) exhibited remarkable antifungal activity against dermatophytes and against *C. neoformans* standardized strains, as well as against a number of clinical isolates. Complementarily, we have carried out a structural molecular and electronic study on compound **5** to reveal the conformational and electronic characteristics of this compound. Predictions of ADME, absorption and distribution parameters and the calculated physicochemical properties (log S = −4.4, clog P = 3.7) for compound **5** and its analogues, are within the typical ranges desired for a drug, as well as the fulfillment of Lipinski's rule permit us to consider this substance as a good lead compound for antifungal activity. All these aspects serve to justify future research on new series of phthalazinones focused on the structural optimization that could lead to a substantial improvement of potency and antifungal activity spectrum. Such research must be complemented with *in vivo* toxicity and efficacy evaluations and the elucidation of the mechanism of action.
